# Yogurt Acid Whey Utilization for Production of Baked Goods: Pancakes and Pizza Crust

**DOI:** 10.3390/foods8120615

**Published:** 2019-11-25

**Authors:** Julie Camacho Flinois, Robin Dando, Olga I. Padilla-Zakour

**Affiliations:** Department of Food Science, Cornell University, Ithaca, NY 14853, USA; jif33@cornell.edu (J.C.F.); rd426@cornell.edu (R.D.)

**Keywords:** acid whey, ingredient, sustainable, baked goods, pizza, pancake

## Abstract

The increased production of Greek-style yogurt in the past decade has induced the need for the reintroduction of the nutrients of its byproduct, yogurt acid whey (YAW), into the food system to combat food waste and aid sustainability. However, the processing and treatment of acid whey, which can be environmentally damaging if disposed of incorrectly, can be costly and complex. Upscaling YAW as an ingredient in food products with minimal re-processing is a cost-effective way to bypass the need for further abatement. To span a broad spectrum of baked products (sweet and savory, biologically and chemically leavened, dairy or water based, oven or surface baked, batter or dough, etc.), pilot commercial pizza crust and pancake formulations incorporating acid whey as a functional ingredient were developed. Dimensions and physico-chemical properties of samples were measured at production and over shelf life at room temperature (23 °C). Consumer sensory testing (*n* = 120 and *n* = 108, respectively, Just About Right (JAR), nine-point hedonic, purchase intent, and demographics) were conducted for both products. All instrumental trials and analyses (°Brix, a_w_, color attributes, viscosity, dimension measurements, and texture analysis) were conducted in triplicate for statistical analysis. Cochran’s Q and post-hoc tests on sensory data showed that liking for at least one experimental YAW sample for each of the pizza and pancake formulations were on par with their respective commercial product, despite the reduction of buttermilk, salt and sugar from the YAW formulations. Adding sustainability claims brought the purchase intent on par with the controls. Replacement of water by weight of YAW was more appropriate than by water content of the YAW. Sourness was the main undesirable trait of YAW samples based on penalty analysis. The use of YAW improved the shelf life of baked goods based on their respective failure mechanisms (textural properties and mold growth). YAW is a suitable ingredient in the formulation of sustainable, healthy, safe, and commercially successful baked products that have a tolerance or can benefit from a sour flavor profile.

## 1. Introduction

Water is a major ingredient used in the processing of baked products. Through its chemical interactions (hydration, dispersion, dissolution, gelatinization, suspension, emulsion, etc. [1]) with other ingredients (proteins, starches, and fats), water has many effects on the physical properties of final products. Water may be added to the formulation directly, as part of a dairy ingredient such as milk or buttermilk (up to 91% water content), or to rehydrate powdered dairy ingredients [1]. Those liquids’ properties such as hardness (mineral content, specifically calcium and magnesium), pH, and microbial load have important effects on the textural, sensory, and shelf life attributes of the final products.

Acid whey is a byproduct of the manufacture of strained Greek-style yogurt and cream cheese. Similar to sweet whey (originating from the manufacture of hard cheeses), it is produced in large amounts (>200,000 metric tons a year [2,3,4]) due to the increasing popularity of its parent product, Greek-style yogurt [5]. Yogurt acid whey (YAW), which contains around 95% water, has a dry mass consisting mostly of lactose and galactose (about 4%), leading to a high biological oxygen demand (BOD) [3,6,7], raising serious environmental concerns [6], and making its disposal a challenge for processors [4,8]. Unlike sweet whey, the starting material for whey protein isolates and concentrates (WPIs and WPCs, respectively) found in many manufactured food products, YAW has not yet reached that level of development. Drying (spray-drying) and membrane separation (micro/ultra-filtration), commonly used for sweet whey, are hindered in the case of YAW by its high acidity and mineral content [3], thus its main current uses are in its raw form as fertilizer and for cattle feed [9,10,11]. Manufacturers are seeking to unlock the potential of acid whey [12], by reintroducing the nutrients it contains into the value-added food chain, or convert them into available energy. This could include fractional concentration to extract valuable ingredients and the generalization of biofuel production [10,13,14]. The discrepancy between the rapid growth of sales of Greek-style yogurt and the availability of technological advances in the processing of YAW call for effective alternative uses in the meantime.

The high water-content and chemical properties of YAW make it an ideal candidate in the baked goods industry, often calling for water and dairy ingredients in large amounts. As a cost-effective resource, YAW provides a dairy flavor, and potential flavor enhancing effects [15]. Its inherent acidity and astringency can be advantageous in products benefiting from such flavor profiles (cheesecake or citrus flavored products, buttermilk cream cheese containing products, etc.).

As an acidic product with pH ranging from 4.21 to 4.48 [16], YAW may be used as a clean-label alternative or complement to added preservatives, in the case of products in which acid flavors are preferred by consumers [17]. The organic acids contained in YAW (lactic acid 0.65%, citric acid 0.18%, and glutaric acid 0.06%) may have antifungal effects [18], increasing product shelf life. The acidity of YAW may contribute to the flavor of fermented doughs and batters. Interactions of the protons in YAW with chemical leavening agents (containing sodium bicarbonate) may lead to faster release of carbon dioxide gas [1], leading to an increase in product yield for short baking time products such as pancakes.

Sodium chloride (table salt) is a recurrent ingredient in baked goods as it strongly affects flavor [19]. The high mineral content of YAW, mainly potassium (>150 mg/100 g), calcium (>120 mg/100 g), and phosphorus (>60 mg/100 g), may help reduce sodium content while maintaining pleasant sensory properties [15], which may be attractive to hypertensive and health-conscious consumers [20]. Mineral salts such as calcium and magnesium are helpful to reinforce the gluten network and are not present in YAW in such high concentrations (>180 mg/L) as to rigidify the gluten structure excessively.

The sugars contained in YAW, mainly lactose (>3%) and galactose (>0.6%), are useful to the baking industry as they can provide a sweet flavor and a softer dough [17]. They are also known to have an effect on Maillard browning [21], a capacity to lower Equilibrium Relative Humidity (ERH), and may also encourage fermentation [17]. These effects may complement those of added sucrose, lowering the use of added sugars in baked products. The water activity of a 16.7% sucrose solution is 0.998 [22], while that of a 16.7% lactose solution is 0.988 [23], showing the higher water-binding potential of lactose compared to sucrose. The decrease of added sucrose, replaced by YAW intrinsic lactose for sweetness, may therefore lead to lower water activity of the final YAW products.

The combined effect of non-sodium salts and non-sucrose sugars to bind water lower the amount of water available to support the growth of microorganisms without resulting in a high added sodium or sugar product.

Containing between 1.7 and 3.7 g/L of protein [16], YAW may help increase product total protein content when compared to the equivalent product manufactured with water while reducing costs for products formulated with dairy powders and modified whey ingredients rehydrated with water.

To assess the feasibility of using YAW in the manufacturing of baked goods, two model products, American-style pancakes and pizza (ready-to-eat and par-baked pizza crust), were chosen. These two products are widely consumed and suitable to span the baked goods category: sweet and savory products, yeast leavened (fermented) and chemically leavened products, baked goods made primarily with water or dairy as the liquid phase, oven-baked and surface-baked products, batter (~150 baker’s% water), and dough (~60 baker’s% water). Both products traditionally contain dairy ingredients and/or modified whey ingredients, and thus no new allergen would be introduced from using YAW. We can therefore postulate that YAW could be a suitable major ingredient (Top 3 by weight, at the traditional level of water or liquid dairy) in the formulation and manufacture of a variety of baked goods.

## 2. Materials and Methods

Ingredients: YAW (Byrne’s dairy, NY, USA) was collected and stored refrigerated (5 °C) for no more than a week before being heated to 93 °C/181 °F for 3 min for pasteurization [24]. Flour (11.7% and 12.7% protein, King Arthur Flour, Norwich, VT, USA), sodium chloride (Morton Salt, Chicago, IL), soybean oil (Wal-Mart Stores, Inc., Bentonville, AR, USA), instant yeast (Lesaffre, Marcq-en-Baroeul, France), purified water (Nestlé S.A, Switzerland), and sucrose (Wal-Mart Stores, Inc., Bentonville, AR, USA) were purchased from a national supermarket in Ithaca, New York. Dextrose and sodium bicarbonate were obtained from J.T. Baker Chemical Company (Phillipsburg, NJ, USA). Defatted Bakers soy flour was obtained from ADM (Chicago, IL, USA). Dried egg yolk was obtained from Sonstegard Food Company (Sioux Falls, SD, USA). Buttermilk powder (raw spray powder, 31%) was obtained from Dana Food Inc. (Hillsboro, WI, USA). Sodium acid pyrophosphate 40 (SAPP 40) and monocalcium phosphate monohydrate (MCP) were obtained from ICL Performance Products LP (St. Louis, MO, USA).

Pancake preparation: Three pancake formulations with acid whey were derived from a standard commercial pancake formula (used as the control sample: PanCo) made with purified water in place of YAW in the experimental samples. Table 1A shows the concentrations of each ingredient for the experimental samples as well as the commercial pancake formula. One experimental sample (PanY6.5) was made by replacing the water by weight with native concentration acid whey (6.5 °Brix), and omitting buttermilk powder, without any other change in the formulation. The other two experimental samples were made by replacing the water with the corresponding water content of acid whey at native concentration (PanY6.5A) and concentrated ~2.5× (15 °Brix) (PanY15A), respectively, based on our previous results concerning the replacement of buttermilk with acid whey [15]. In these two experimental samples, buttermilk powder was also omitted, and sugar and salt were lowered proportionally to the sweetening and salting power of the corresponding acid whey. For native concentration, salting power was approximated by the total mineral content (total of 0.4%YAW of calcium, sodium, phosphorus, potassium, and magnesium [16]) while sweetening power was calculated as the product of the amount of sugars (3.3%YAW lactose and 0.59%YAW galactose [16]) by their corresponding sweetening power compared to 1 for glucose (0.16 and 0.6, respectively [25]). The water content of the YAW (94.5% for native and 87.5% for concentrated) were obtained following the oven drying method (sand pan technique at 100 °C) described in Pegg, Landen, and Eitenmiller [26].

Pancake formulation and procedure were adapted from the method by Finnie, Bettge, and Morris (1996) [27]. The dry ingredients (sucrose, dextrose, sodium chloride, soy flour, dried egg yolk, sodium bicarbonate, SAPP 40, MCP, and dried buttermilk if present) were weighed, combined, and mixed by hand for 1 min with a wire whisk for visual homogenization. Flour was then incorporated and mixed for an additional 1 min. The liquid (purified water, native acid whey, or concentrated acid whey) and the liquid vegetable shortening, both at room temperature (23 °C), were added to the dry blend in a stainless-steel mixing bowl and mixed by hand for 30 s with a wire whisk. The resulting batter was then rested for 3 min before measuring its viscosity and baking.

Three minutes after the incorporation of liquids into the dry pancake-mix, a 3 Tbsp/45 mL/1.5 oz stainless-steel commercial scoop with mechanical sweep (Norpro 704 Grip-EZ, Everett, WA, USA) was used to pour equal volumes of batter onto a 20″ ceramic electric commercial griddle (Wal-Mart Stores, Inc., Bentonville, AR, USA) with a surface temperature of 190 °C (±5 °C). The batter aliquots were distributed onto the griddle from a height of ≈8 cm following the aforementioned method [27]. After 75 s of baking on the first side, pancakes were flipped and baked for another 75 s on the other side. Ten pancakes of each of the 12 batches (triplicates of each of control and three experimental formulas) were made. The pancakes were placed on a wire rack, and first cooked side up until cooled below 25 °C. Each was then stored in sterile digester bag (Nasco WHIRL-PAK, Milton WI, USA), heat sealed twice at 1 cm distance, and stored in a dark at room temperature (23 °C) for accelerated shelf life study.

Samples were prepared three times: (1) PanCo, PanY6.5, PanY6.5A, and PanY15A samples were prepared using unpasteurized YAW (YAW stored in clean containers from the time of collection, and kept for seven days refrigerated) for texture analysis; (2) samples were prepared using pasteurized YAW for consumer sensory testing; and (3) samples were prepared using pasteurized YAW for physical analysis and textural and shelf life study. Sample PanY15A was excluded from the third phase based on the consumer sensory testing results.

Pizza and par-baked pizza crust preparation: Two pizza formulations with acid whey were derived from a standard commercial pizza formula made with purified water used as the control (PzzCo). Table 1B shows the concentrations of each ingredient for the experimental samples as well as the commercial pizza crust formula. One experimental sample (PzzY6.5) was made by replacing the water by weight with native concentration acid whey (6.5 °Brix) without any other change in the formulation. The other experimental sample (PzzY6.5A) was made by replacing the water by the corresponding water content of acid whey at native concentration (6.5 °Brix) and lowering sugar and salt proportionally to the sweetening and salting power of the acid whey, calculated as stated above.

The dry ingredients (sucrose, flour, and yeast) were weighed, combined, and mixed in a stand mixer with 3.5 quarts stainless steel bowl and hook extension (KitchenAid, Benton Harbor, MI) for 3 min on the lowest setting. Liquid (purified water, native acid whey or concentrated acid whey) at 35 °C/95 °F was then incorporated and mixed for 7 min on the third setting. Sodium chloride and shortening were added and kneaded in for an additional 6 min on the second setting. The batter was then left partially covered for a 30 min “open-proof” at room temperature, before being separated into 50 g dough balls. The balls were sheeted by passing through a pasta roller (Weston, Southern Pines, NC, USA) 10 times with 5 mm spread between the two rolls to ensure even thickness. Dough sheets were then poked with a hand dough docker (Orblue, Miami FL, USA). Ten mini-par-baked-pizza crusts were made for each of nine batches (triplicates of the control and both experimental pizza crusts) and baked for 5 min at 450 °C turning trays around 180° half-way through for even baking. After letting the crusts cool to below 25 °C, each was stored in sterile digester bag (Nasco WHIRL-PAK, Milton, WI, USA), heat sealed twice at 1 cm distance, and stored in a dark at room temperature (23 °C) for accelerated shelf life study. For sensory evaluation, 350 g dough balls were stretched using the same method to rectangles of 15 cm × 45 cm and docked. Half were covered in marinara sauce (Barilla, Parma, Italy) and mozzarella cheese (Great-value, Wal-Mart Stores, Inc., Bentonville, AR, USA). Samples were baked for 12 min at 450 °C flipping trays half-way for even baking. All rectangles were trimmed on each short side by 2.5 cm, cut in half longitudinally once and vertically at 5 cm interval to produce 16 equal-sized (7.5 cm × 5 cm) pieces with one edge.

Sensory evaluation: All procedures were reviewed and approved by the Cornell Institutional Review Board for Human Subjects Research, with all panelists providing informed consent. In the first Central Location Tests (CLT), the four pancakes were served (at room temperature, 23 °C) in a random (counter-balanced design) and blinded consumer study (*n* = 120) in a monadic sequential manner [28]. On a separate day, the three pizza crusts were served (warm) in a randomized and blinded consumer study (*n* = 108) in a monadic sequential manner, followed by all three samples of pizza served together for direct comparison. During the direct comparison stage of the pizza sensory study, a ranking of the three samples was requested as well as a score on the Labeled Affective Magnitude (LAM) scale for each, featuring a 100-point scale from greatest imaginable dislike (100) to greatest imaginable like (0). Demographic data were collected from the panelists in each test, with sensory data collected using the nine-point hedonic scale for overall, appearance, texture, color, and flavor liking. Just About Right (JAR) scales were used for texture and flavor attributes, as well as questions on purchase intent (before and after giving information about the sustainability claims), preferential ranking, and short open-ended questions also asked after liking. Testing was performed at the Cornell Sensory Evaluation Facility, in individual booths, under standard lighting. Panelists for both tests were users of the category (consuming the product in question at least a few times a year) and recruited with normal senses of smell and taste and with no salient food allergies. Sensory data and sensory data statistical analyses were gathered using RedJade (Curion Insights, Redwood City, CA, USA).

Phisico-chemical evaluation: Before baking, batter viscosity of the pancake was measured. Pancake batter was poured into the reservoir of a Bostwick Consistometer (CSC Scientific Co., Fairfax, VA, USA) and excess batter was scraped off the top of the reservoir with a flat metal blade. The consistometer gate was released, and after 1 min the distance (in cm) that the batter traveled was recorded as “Bostwick” batter viscosity.

Each day after production (Day 0), one sample was selected randomly for each of the triplicate batches of the four pancake samples and three pizza crust samples. The diameter of each was measured with a digital caliper (Fisher Scientific) at three points on the pancake (smallest, largest: 0°, and 90°) and averaged. Aliquots of 4 cm diameter were cut out of each sample. On Day 1, one aliquot was randomly selected to observe the gas cell structure by peeling off the top layer. Three aliquots were measured for color components (UltraScan VIS, HunterLab, VI, USA) on Days 2–4. Three aliquots were used on Day 2 for moisture content analysis following the oven drying method at 70 °C, as prescribed for samples high in carbohydrates by Pegg et al., 2010 [26]. The thickness of each aliquot was measured with a digital caliper at three points (thinnest, thickest, and center) and averaged. Each aliquot was then subjected to TPA (texture profile analysis) using a TA-XT2 Texture Analyzer (Stable Micro Systems, Texture Technologies Corp. Scarsdale, NY, USA) equipped with a 50 kg load cell and a round 75 mm diameter compression platen probe [29,30]. Each aliquot was placed under the probe (first baked side up for the pancake, top side up for the pizza crusts), and compressed to 50% of original height at a constant speed of 1 mm/s. After the initial compression, the probe withdrew for 5 s, followed by a second compression of 50% of the original height. The computer software Texture Exponent 32 (Stable Micro Systems) was used both for the experimental phase and to compute textural parameters from the TPA curve.

Shelf life evaluation: Given the difference in failure mechanisms, shelf life was assessed separately for pancakes and pizza crusts. In addition to the TPA recorded over five days, preliminary shelf life evidence was recorded using visible mold growth. Mold free shelf life of at least 12 units was recorded for each pancake sample over eight days after production. Three pizza crusts samples were set aside from each batch (*n* = 9) and the surface covered by mold was recorded as a percentage of the total surface using imageJ software over eight days after production.

Statistical analysis: Throughout the study, samples were prepared in triplicates for instrumental testing, and measurements were performed in triplicates for each sample, leading to at least nine individual observations for each variable for each condition. Statistical data analyses (one-way ANOVA for physical data, two-way ANOVA for sensory data, and Cochran’s Q post-hoc test) were performed using IBM SPSS Statistics (version 21, IBM Corporation, Armonk, NY USA). For hypothesis testing, a significance level of *p* < 0.05 was used.

## 3. Results and Discussion

### 3.1. Effect of YAW Content on Physical Properties of Baked Goods

**Figure 1:**
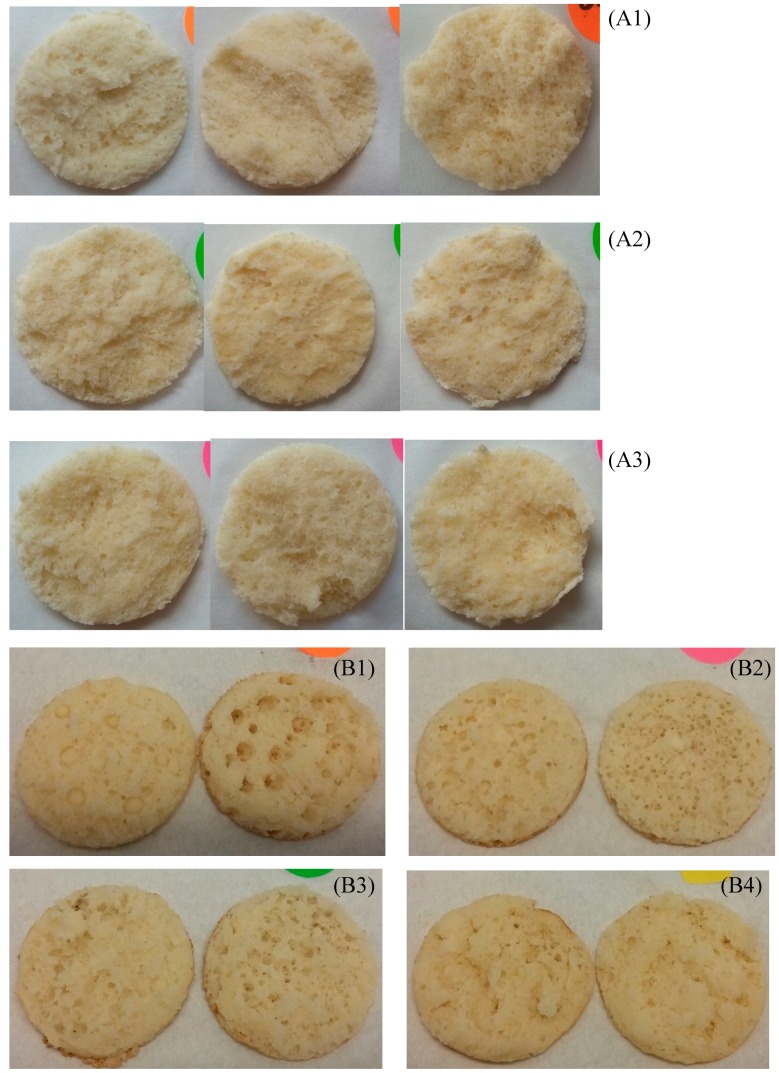
Crumb structure of the negative control pizza (Pizza made with water and control formula): (**A1**); Positive control pizza (Pizza made with native concentration YAW with no change to the formula) PzzY6.5 (**A2**); and experimental pizza (Pizza made with native concentration YAW and with formula modified to account for salts and sugars in the YAW: PzzY6.5A) (**A3**); and the negative control pancake (Pancake made with Water and the original pancake formula): (**B1**); the positive control pancake (Pancake made with YAW at native concentration and the original pancake formula: PanY6.5) (**B2**); the first experimental pancake (Pancake made with YAW at native concentration and modifications to the formula to account for the sugars and salts in the YAW: PanY6.5A) (**B3**); and the second experimental pancake (Pancake made with YAW concentrated to 15 brix and modifications to the formula to account for the sugars and salts in the YAW: PanY15A) (**B4**).

The use of whey minerals in breads to replace sodium from table salt showed favorable results in a previous study with decreased bread density when using 2% whey minerals instead of 2% table salt [31]. In our results, the density of the par-baked pizza crusts did not decrease, with a higher density for PzzY6.5 (coherent with a lower moisture content) and a similar density between control and PzzY6.5A (Table 2). Par-baked pizza crust samples made with YAW seemed to have a stronger gluten structure. This can be seen in the smaller diameter (PzzY6.5A < PzzY6.5 < PzzCo) due to the retraction of the dough during baking; a higher thickness (PzzY6.5A > PzzY6.5 > PzzCo), reflecting a high capacity to retain gas formation in crumb cells; and an aerated crumb structure (Figure 1A). The gluten network within a fermented dough is developed during kneading time and is influenced by the minerals present in the dough leading to its specific rheological properties [32]. On the other hand, high levels of salts (including added NaCl) restrain yeast activity in fermented doughs, thus mineral salts present in YAW and added to the dough may have the same effects. The marginally lower volume of the experimental samples is not observed again in the cell structure of samples (Table 2). The use of YAW in bread doughs may allow for lower kneading energy requirements than the corresponding dough made with water to reach the desired gluten development and potentially thinner sheeting to decrease the height to diameter ratio. Higher levels of yeast or longer floor time may help increase total fermentation to maintain total volume.

The reaction of the acids present in YAW with the chemical leavening agent (sodium bicarbonate), leading to a faster release of carbon dioxide gas in the pancake batter [17], would explain the lower density of PanY6.5 (0.399 g/cm^3^) compared to control (0.503 g/cm^3^) (Table 2). This early release of carbon dioxide is beneficial to the volume of pancakes in the case of a higher viscosity batter (PanY6.5, ~10 Bostwick score like control), but detrimental in the case of a lower viscosity batter (PanY6.5A, ~15 Bostwick score), which limited gas retention through the baking period, leading to a higher density of the final product (PanY6.5A: 0.707 g/cm^3^). The crumb structure of pancake samples revealed larger, less numerous gas pockets in the control than in PanY6.5, and the smallest gas cells in PanY6.5A (Figure 1B). In the case of the Pizza crumb structure (Figure 1A) no noticeable difference was detected.

The Maillard reaction, responsible for many attractive aromas in baked goods as well as a desirable coloration, renders to a darker (lower L* value) and more yellow (higher b* value) bread [33]. In the case of the par-baked pizza crusts, no significant color difference was detected, and color liking (one-way ANOVA, *p*-value = 0.38) was not significantly different for the three samples tested. For all the pancakes, both experimental samples had a higher L* (lighter) and b* (yellower) values and lower a* (less red) values than the control (Table 2). When compared to results of consumer testing results, color liking was also significantly higher for PanY6.5 (7.10) followed by PanY6.5A (6.70) and control (6.47). Pancakes made with YAW were more attractive visually due to the deeper yellow color and thus may have a higher tolerance for increased baking time or temperature (which would decrease L* to the level of the control samples.

### 3.2. Effect of YAW Content on Shelf Life of Baked Goods

The speed of microbial spoilage on bakery products is determined by the microbial load of the ingredients, the environmental conditions (microorganisms present, moisture, and temperature), the product’s surface water content, water activity, pH, and the presence of mold inhibitors [17]. The shelf life test of all samples was conducted for eight days at room temperature (23 °C) to observe major changes in quality due to the different formulations. Since samples were packaged in sealed, sterile bags, apparent differences in shelf life between control and experimental samples can be due to water activity, moisture content, microbial load, product pH, or antimicrobial compounds. All of these characteristics can be directly related to the inclusion of YAW or the changes in formulation related to the addition of YAW for water, sugar, and salt content adjustments.

Pancake and par-baked pizza crusts made for this study with a moisture content of 33.08–54.42% are representative of the average moisture content of intermediate moisture range baked goods [17].

**Figure 2:**
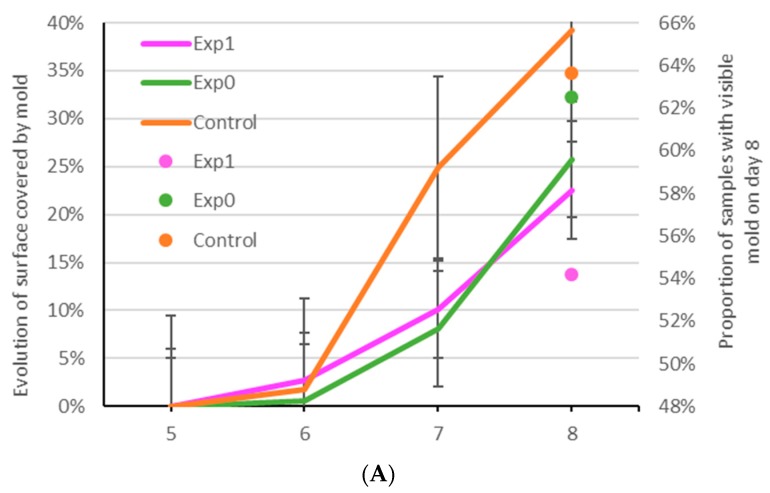

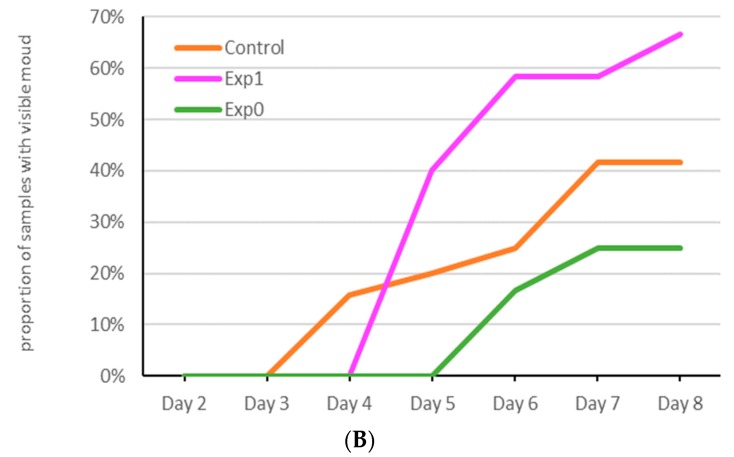
Shelf life study of par-baked pizza crusts and pancakes stored at room temperature (23 °C) in the dark in individual aseptic packaging: total surface covered by mold on Days 5–8 and proportion of samples presenting visible mold colonies on Day 8 of par-baked pizza samples made with and without YAW (**A**); and number of samples with at least one visible mold colony on pancakes samples between Days 2 and 8 (**B**).

All experimental samples made with pasteurized YAW exhibited a higher proportion of mold-free samples by Day 8 (Figure 2), coherent with the presence of lactic acid in YAW. In the case of the pancakes, the proportion of mold-free samples was higher in PanY6.5, which has a lower moisture content (50% vs. 53% for the control) and lower water activity (by around 0.01). However, at the same water content of 53% and the same water activity of 0.966, the control sample had a faster mold-growth onset but a slower increase of the percentage of spoiled samples than PanY6.5A. Visible mold colonies appeared on the experimental samples on Days 4 and 5 for PanY6.5A and PanY6.5, respectively, compared to Day 3 for control samples (Figure 2B), suggesting a longer “mold-free shelf life” of experimental samples. The strong putrid smell of control pancake samples and the moist, sticky, and stringy nature of the crumb by Day 5 indicated that the microbial growth was not limited to mold and that the failure mechanism for the pancakes may be bacterial [34,35]. Thus, the control pancake samples had a shorter shelf life than presumed from mold growth observations, placing it second with 42% of samples presenting visible mold against 25% for PanY6.5 and 66% for PanY6.5A (Figure 2B).

In the case of the par-baked pizza crusts, both experimental samples performed better than the control in terms of speed of mold growth (based on the total surface of pizza samples covered by mold) with a lower percentage of mold surface area (22–27% for PzzY6.5 and PzzY6.5A and 38% for control) and lower or similar number of samples with visible mold by Day 8 (54% for PzzY6.5 and 63–64% for PzzY6.5A and control), for the same onset at Day 5 (Figure 2A). These improved shelf life results were irrespective of their lower (32% for PzzY6.5) and equal (33% for control and PzzY6.5A) moisture content at the same water activity of 0.95 (all samples).

Our results indicate that the use of YAW may reduce microbial growth, and therefore provide shelf life extension of some baked goods in formulations without added preservatives.

**Figure 3:**
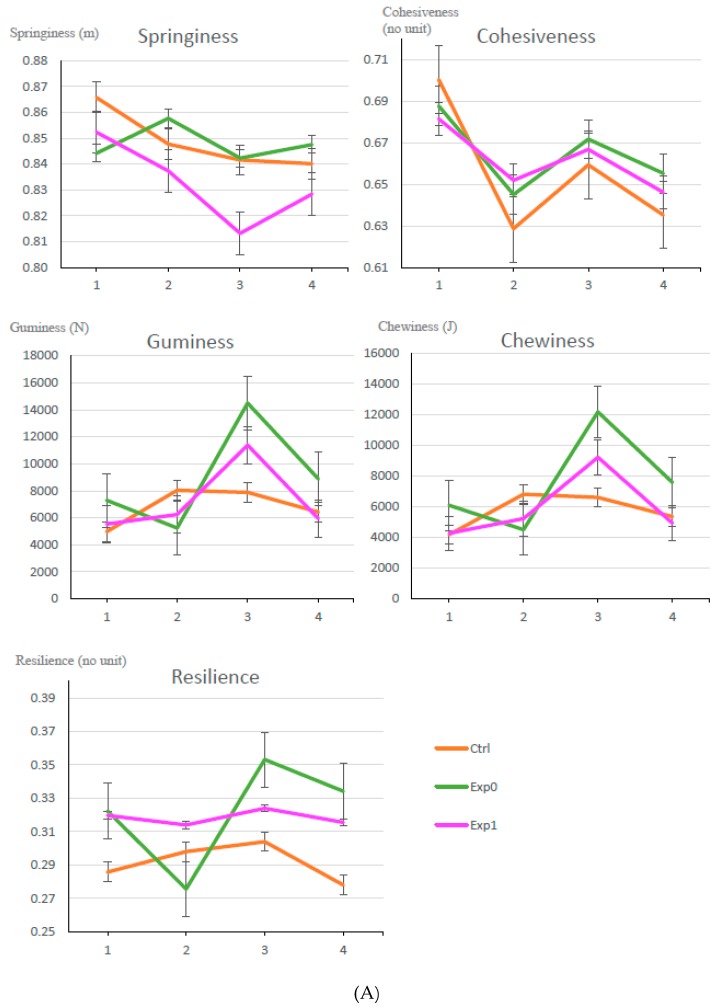

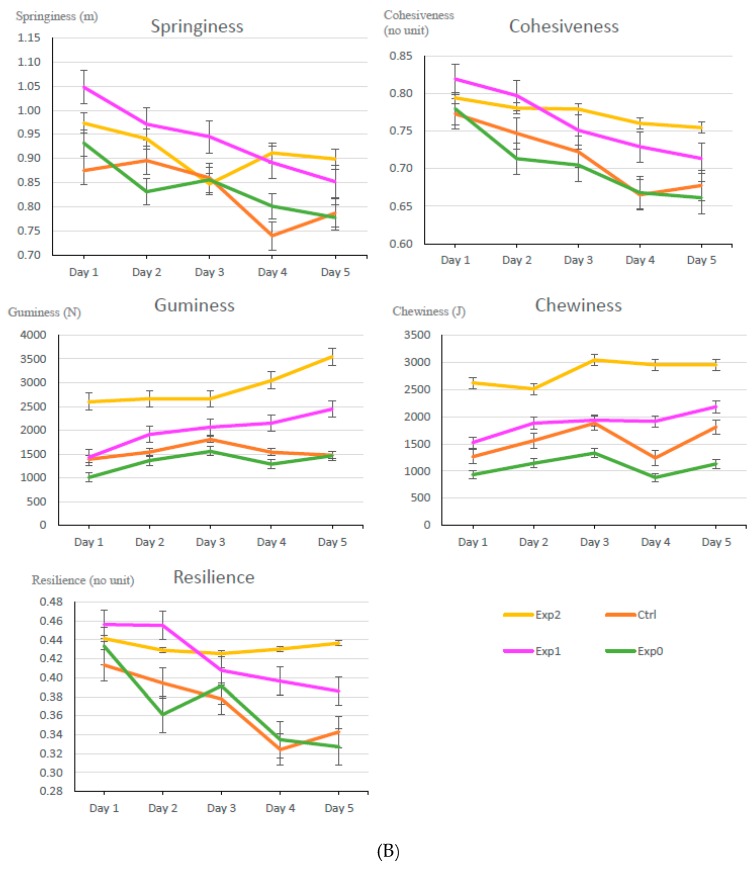
Textural analysis (TPA) of: the control pizza (Ctrl), PzzY6.5 (Exp0), and PzzY6.5A (Exp1) (**A**); and the control pancake (Ctrl), PanY6.5 (Exp0), PanY6.5A (Exp1), and PanY15A (Exp2) (average of similar unpasteurized and pasteurized data) (**B**).

All textural attributes seemed to be similar between the experimental and control samples (Figure 3) even though PzzY6.5 and PanY6.5 had lower moisture content. Textural properties of par-baked pizza samples did not change significantly across the five-day period, confirming that the failure mechanism off the par-baked pizza shelf life was not textural but microbiological. The pancake samples showed significant differences in texture for the PanY15A and PanY6.5A compared to PanY6.5 and control, showing stronger values. The increase in gumminess over the storage time of all pancake samples may be indicative of bacterial spoilage.

### 3.3. Effect of YAW Content on Sensory Attributes of Baked Goods

All panelists participating in the studies were frequent consumers of pancakes (consuming them few times a year (37%), every month (48%), or every week (15%)) and pizza (consuming pizza a few times a year (12%), every month (52.8%), or every week (35.2%)). The panel was 71% female and 29% male, and 8% over 55 years old, 57.5% 23–54 years old, and 34.5% under 22 years old.

Overall liking of the pancakes was on par (two-way ANOVA, *p* > 0.05) between the control 6.4 (±1.4) and PanT6.5 6.3 (±1.6). PanY6.5 also received significantly higher overall appearance liking (7.1 ± 1.3 compared to 6.7 ± 1.5 for control, two-way ANOVA post hoc *p*-value < 0.05), color liking (7.1 ± 1.4 compared to 6.5 ± 1.8 for control), and textural-appearance liking scores (7.2 ± 1.3 compared to 6.8 ± 1.6 for control). Dairy flavor JAR score was equal for the control and all experimental samples (averaging 3.70), suggesting that the removal of buttermilk powder from the formulation without losing the dairy flavor was achievable when using YAW instead of water. All sensory measures of texture were equivalent between the control and PanY6.5 (texture liking 6.5 ± 1.8 and 6.5 ± 1.9, fluffiness JAR 3.9 ± 0.7 and 3.8 ± 0.6, chewiness 4.4 ± 0.8 and 4.4 ± 0.8, moistness 3.9 ± 0.7 and 3.9 ± 0.7, and softness 4.1 ± 0.6 and 4.0 ± 0.4), despite a 3.5% lower moisture content (51% for PanY6.5 compared to 54.5% for the control, Table 2). Moisture content of pancakes, similar to other cake products, is a key factor in the consumer’s perception of freshness and therefore product quality [36]. The replacement of water and buttermilk powder in batters with YAW was best achieved with a per-weight replacement of water with YAW (rather than a per-water-weight replacement, leading to texture JAR scores different from control for PanY6.5A) for the retention of perceived textural properties on a lower moisture content product. These results are consistent with TPA analysis conclusions that YAW allows for similar textural properties (physical and sensorial) at a lower moisture content of the final product.

**Figure 4:**
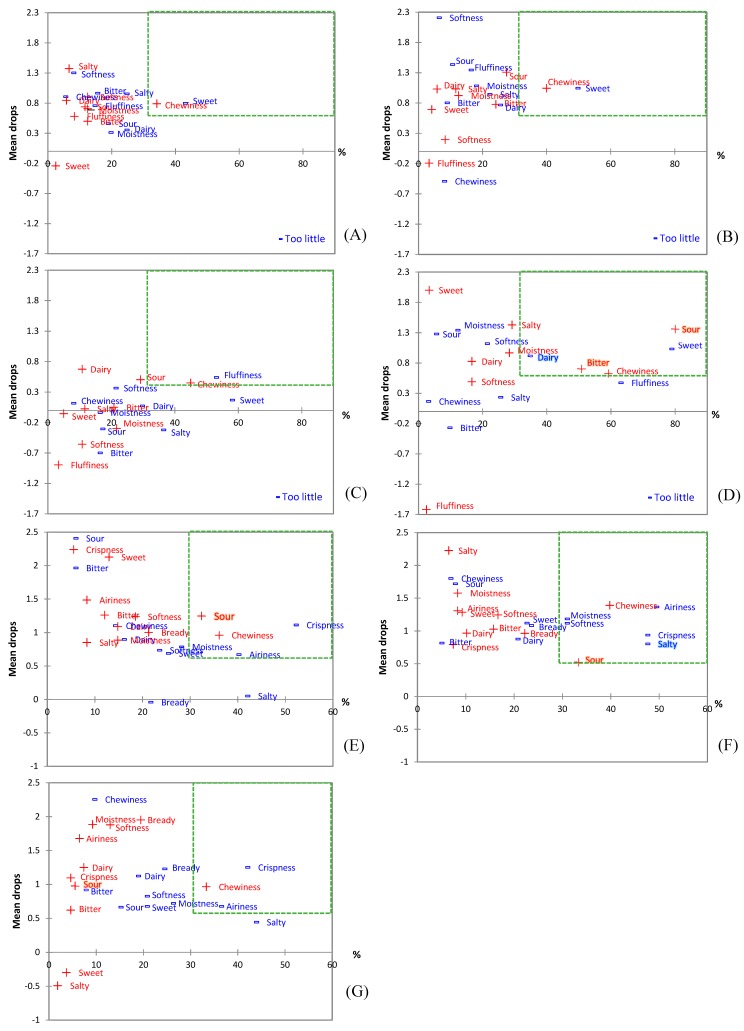
Penalty analysis (mean drop vs. percent): of the control pancake (**A**); PanY6.5 (**B**); PanY6.5A (**C**); PanY15A (**D**); PzzY6.5 (**E**); PzzY6.5A (**F**); and the control pizza (**G**).

According to penalty analysis (Figure 4), the lower overall liking score of PanY15A was mainly due to the increased bitterness and sourness brought by YAW. These results coincide with the lower scores of PanY15A for appearance, color, texture, and aroma liking; the strongest aroma intensity; and 68% of panelists reporting an aftertaste (compared to 11% for the control, 18% for PanY6.5, and 15% for PanY6.5A), leading to the exclusion of PanY15A from further testing. For pizza, PzzY6.5 received a higher flavor intensity score (3.8 ± 1.0) than its respective control (3.3 ± 1.0); otherwise, all other experimental samples received a similar flavor intensity as their respective control (despite the removal of buttermilk powder in the case of pancakes). These results are in agreement with the potential flavor enhancing effects of YAW as an ingredient demonstrated in our earlier study on Ranch dressing [15,36].

Penalty analysis (Figure 4) showed similar results in the case of pizza crust samples to the pancakes, with a lower overall liking score (PzzY6.5 (5.5 ± 1.7) and PzzY6.5A (5.4 ± 1.9) compared to the control (6.3 ± 1.6)) caused by the increase in sourness and decrease in saltiness. The purchase intent of pizza crusts was also lower for experimental samples (2.8 ± 1.0 for PzzY6.5 and 2.8 ± 1.1 for PzzY6.5A) than for the control (3.2 ± 1.1), accompanied by a higher percentage of panelists detecting an aftertaste (19% and 14%) in experimental samples than in the control (5%). This lower overall liking score of the crust alone did not translate to a decreased liking of the final pizza product (*p* = 0.46, ranked sum test), with a similar overall liking score on the LAM scale: 32.2 ± 14.0 for the control, 34.1 ± 15.5 for pizza made with PzzY6.5 crust, and 32.3 ± 14.8 for pizza made with PzzY6.5A crust.

Furthermore, purchase intent (Table 3) was collected once for all samples without information, and a second time after informing the panelists that samples are “made with upcycled ingredients from the dairy industry which avoids pollution from dumping nutrients in water streams, and reduces food waste by reintroducing nutrients in food products rather than disposing of them”. The purchase intent of the experimental pizza crusts after informing the panelists of the sustainable nature of the product were not different (one-way ANOVA, *p*-value: 0.0968) from that of the control. Similar results were obtained for pancakes PanY6.5A and PanY6.5, receiving purchase intent scores not different (one-way ANOVA *p* > 0.05, post Hoc *p*-value < 0.05) from that of the control after stating their appropriate sustainability claim.

Nutritionally, an increase in Vitamin D (+2 to 8% Recommended Daily Value (RDV)), calcium (+4 to 13% RDV), and potassium (+2 to 4% RDV) can be observed for all samples containing YAW. The decrease in added sugars and added sodium in the formulations for PanY6.5A, PanY15A, and PzzY6.5A is evident in the nutrition labels (Table 4), lowered by 0.5–1 g/serving and 55–110 mg/serving for added sugar and sodium, respectively, between PzzY6.5A and PanY6.5A and their respective controls. These decreases did not translate to any drop in sweetness or saltiness perception in pizza: on the seven-point JAR scale, saltiness perceptions was rated 3.4 (±0.9) for the control, 3.5 (±1.0) for PzzY6.5, and 3.4 (±0.9) for PzzY6.5A, and sweetness was rated as 3.8 (±0.6) for the control, 3.8 (±0.8) for PzzY6.5, and 3.8 (±0.7) for PzzY6.5A. However, these improvements on the nutrition facts panel did translate in a sweetness and saltiness decrease in the case of PanY15A pancakes: on the seven-point JAR scale, saltiness perceptions were rated 3.7 (±0.9) for the control, non-significantly different from 3.9 (±0.9) for PanY6.5 and 3.6 (±1.0) for PanY6.5A, but 4.1 (±1.3) for PanY15A was significantly higher. Similarly, sweetness perceptions were rated as 3.5 (±0.8) for the control, not different from PanY6.5 (3.4 (±0.9)), but significantly higher than 3.2 (±1.0) for PanY6.5A and 2.7 (±1.1) for PanY15A. This suggests that YAW minerals and native sugars can replace added sucrose and salt nutritionally and perceptually to a certain extent, possibly up to total mineral amount and up to sweetness power (as defined above as the sweetness equivalent of glucose) in the case of breads (~60 baker’s% water content) but to a lower extent for cakes (>150 baker’s% water content), especially for sucrose replacement.

## 4. Conclusions

YAW use as a per-weight basis replacement of water may lead to an increase in protein, calcium, and potassium and a decrease in sodium and added sugars, while conserving flavor. Textural attributes, a common sign of a baked product’s freshness, are conserved in YAW-based products even at a lower moisture content. In the case of baked goods, the ingredient list is unlikely to be extended by the introduction of YAW compared to products currently available on the market; it may even be shortened by removing acids, salt, sugar, dairy ingredients such as buttermilk, or added dairy flavors. Additives and preservatives may also be omitted, taking advantage of the shelf-life extension provided by YAW. This elimination of ingredients from the list is accompanied by an equivalent subtraction of the cost of these ingredients, which can provide a competitive advantage to the manufacturer and benefit the consumer.

## Figures and Tables

**Table 1:** (A) Formulation of Pancakes: Ingredients as a percentage (%) and percentage of flour (Baker’s %). (B) Formulation of Pizza crusts (Ingredients as a percentage of flour, Baking %).

**(A)**								
**Ingredients**	**Control**		**Experimental 0**		**Experimental 1**		**Experimental 2**	
	**Baker’s%**	**%**	**Baker’s%**	**%**	**Baker’s%**	**%**	**Baker’s%**	**%**
Sucrose	10.67	3.55	10.67	3.59	9.33	2.92	6.97	1.97
Dextrose	3.33	1.11	3.33	1.12	3.33	1.04	3.33	0.94
Sodium Chloride	0.67	0.22	0.67	0.22	0.00	0.00	0.00	0.00
Soy Flour	6.33	2.11	6.33	2.13	6.33	1.98	6.33	1.79
Egg Yolk Powder	1.33	0.44	1.33	0.45	1.33	0.42	1.33	0.38
Sodium Bicarbonate	2.00	0.67	2.00	0.67	2.00	0.63	2.00	0.56
Sapp40	2.80	0.93	2.80	0.94	2.80	0.88	2.80	0.79
MCP	1.30	0.43	1.30	0.44	1.30	0.41	1.30	0.37
Flour	100	33.28	100	33.62	100.00	31.34	100.00	28.22
Buttermilk Powder	1.33	0.44						
Shortening	3.33	1.11	3.33	1.12	3.33	1.04	3.33	0.94
**Purified water**	167.43	**55.71**						
**YAW (6.7 Brix)**			165.71	**55.71**	188.31	59.33		
YAW Water			155.61	52.31	177.77	**55.71**		
YAW Solids			10.11	3.40	11.55	3.62		
Salting power			0.66 (total minerals)			0.76 (total minerals)		
Sweetening power			6.49 (total sugars)			1.34 (sugars relative sweetness)		
**YAW (15.4 Brix)**							225.55	63.91
YAW Water							268.40	**55.71**
YAW Solids							30.35	8.60
Salting power						(total minerals) 2.08		
Sweetening power						(sugars relative sweetness) 3.70		
**(B)**								
**Ingredients**	**Control**		**Experimental 0**			**Experimental 1**		
	**Baker’s %**	**%**	**Baker’s %**		**%**	**Baker’s %**		**%**
Flour	100	57.64	100		57.64	100		56.07
Sodium Chloride	1.5	0.86	1.5		0.86	1.24		0.69
Sucrose	3	1.73	3		1.73	2.42		1.36
Shortening	8	4.61	8		4.61	8		4.49
Yeast	1	0.58	1		0.58	1		0.56
**Purified water**	60	34.58						
**YAW (6.7 Brix)**			60		**34.58**	65.7		36.84
YAW Water			56.34		32.47	61.69		**34.58**
YAW Solids			3.66		2.11	4.01		2.25
YAW Salting power	(total minerals) 0.24				(total minerals) 0.26			
YAW Sweetening power	(total sugars) 2.352				(sugars relative sweetness) 0.58			

Values in red represent the two ways water can be replaced in the formulas: either by liquid weight replacement between water and YAW or by total water content in the formula since YAW contains water and solids. Values in red and bolded are the variables chosen as constants to the control in each experimental samples. Values in grey represent the corresponding sucrose- or salt-equivalent of sweetness and saltiness power of the minerals and sugars in the YAW added into each formula.

**Table 2:** Physical attributes (color, water activity, dimensions, moisture content, and batter viscosity) of par-baked pizza crust (**A**) and pancakes (**B**) samples formulated with or without acid whey.

	Pizzas (A)			Pancake (B)		
	Control	Experimental		Control	Experimental	
		PzzY6.5	PzzY6.5A		PanY6.5	PanY6.5A
Color (*n* = 27)						
L *	75.3 (±1.7)	76.2 (±1.4)	74.4 (±1.4)	55.6 (±12.0)	72.6 (±5.9)	74.0 (±4.8)
a *	1.7 (±0.1)	1.6 (±0.2)	1.5 (±0.2)	10.5 (±3.1)	2.6 (±3.3)	3.5 (±2.8)
b *	15.4 (±0.6)	16.7 (±0.6)	16.3 (±0.5)	22.1 (±1.6)	28.8 (±5.8)	28.0 (±4.0)
Water activity (aw, *n* = 9)	0.951 (±0.010)	0.946 (±0.000)	0.950 (±0.010)	0.966 (±0.012)	0.958 (±0.006)	0.966 (±0.005)
Moisture (%, *n* = 9)	33.08 (±0.83)	31.78 (±0.46)	33.18 (±0.98)	53.30 (±0.80)	49.72 (±0.36)	52.67 (±0.83)
Dimensions (*n* ≥ 12)						
Height (mm)	11.1 (±1.5)	11.4 (±2.0)	11.7 (±1.6)	11.6 (±1.0)	12.3 (±1.1)	9.2 (±0.8)
Diameter (mm)	103.6 (±6.3)	97.1 (±5.5)	95.9 (±5.5)	81.0 (±16.7)	96.5 (±6.3)	86.7 (±15.9)
Weight (g)	41.5 (±0.1)	45.9 (±0.1)	40.8 (±0.2)	30.2 (±3.1)	35.8 (±3.1)	38.2 (±3.2)
Volume (mm^3^)	93214	84603	84645	59919	89722	54033
Density (g/cm^3^)	0.445	0.543	0.482	0.503	0.399	0.707
Viscosity (*n* = 3)				9.17 (±0.58)	9.83 (±0.38)	14.83 (±0.76)

**Table 3:** Purchase intents of all samples before and after informing panelists of the sustainability claims of the samples.

	Pizzas			Pancakes			
Purchase Intent	Control	Experimental		Control	Experimental		
		Y6.5	Y6.5A		Y6.5	Y6.5A	Y15A
Without Sustainability claim	3.24 (±1.09) AB	2.81 (±1.05) C	2.78 (±1.11) C	3.25 (±0.99) AB	3.17 (±1.03) B	2.75 (±1.02) C	1.84 (±0.99) D
With sustainability claim	3.32 (±1.11) A	3.06 (±1.12) AB	2.92 (±1.08) B	3.54 (±1.14) A	3.44 (±1.18) AB	2.99 (±1.18) B	1.94 (±1.11) D

Data in black represents the purchase intent value of each sample when presented with their appropriate corresponding claim (sustainable product, or no claim). Data in grey shows purchase intent value of the samples when paired with an incorrect sustainability claim (control samples) or paired with no claim when sustainability claim would be warranted (experimental samples). Thus data in black should be compared to each other. Letters represents groups of statistical significance when performing Cochran's Q post Hoc test on the data set.

**Table 4:** Nutritional information (as would appear on a 2016 Nutrition fact panel) for control and experimental Pizza crusts and pancakes. formulations.

	Pizzas			Pancakes			
	Control	Experimental		Control	Experimental		
		PzzY6.5	PzzY6.5A		PanY6.5	PanY6.5A	PanY15A
**Calories**	250	260	250	160	160	150	130
**Total Fat**	4.5	4.5	4.5	1.5	1.5	1.5	1
Saturated Fat	0.5	0.5	0.5	0.5	0.5	0	0
Trans Fat	0	0	0	0	0	0	0
**Cholesterol**	0	0	0	10	10	10	10
**Sodium**	335	350	280	650	670	540	530
**Total Carbohydrate**	42	44	43	31	31	28	25
Dietary Fiber	2	2	2	1.5	1.5	1.5	1
Total Sugars	2	3	3	5	7	6	9
Added Sugars	2	2	1	4.5	5	4	3
**Protein**	7	9	8	6	7	6.5	8
Vitamin D	0%	2%	2%	0%	4%	4%	8%
Calcium	0%	4%	4%	2%	6%	6%	15%
Iron	15%	15%	15%	10%	10%	10%	10%
Potassium	0%	2%	2%	2%	4%	4%	6%
